# Dyskinesia and impulsive compulsive behaviour in Parkinson’s disease are not related: Insights from a study with a wearable sensor

**DOI:** 10.1016/j.parkreldis.2023.105813

**Published:** 2023-10

**Authors:** Lucia Ricciardi, Andrea De Angelis, Chiara Siri, Malcom Horne, Alison Leake, Dominic Paviour, Priyanka Pradhan, Mark Edwards, Francesca Morgante

**Affiliations:** a Neurosciences Research Centre, Molecular and Clinical Sciences Research Institute, St George's University of London, London, UK; b Parkinson Institute, ASST G. Pini-CTO, Ex ICP, Milan, Italy; c Centre for Clinical Neurosciences and Neurological Research, St Vincent's Hospital, Melbourne, Australia; d Florey Institute for Neuroscience and Mental Health, University of Melbourne, Parkville, VIC, Australia; e Institute of Psychiatry, Psychology and Neuroscience at King's College, London, UK; f Department of Experimental and Clinical Medicine, University of Messina, Messina, Italy

**Keywords:** Dyskinesia, Impulse control disorders, Impulsive compulsive behaviors, Parkinson's disease, Wearable sensors, Impulsivity

## Abstract

**Introduction:**

Previous studies have suggested an association between Impulsive Compulsive Behaviour (ICB) and dyskinesia in Parkinson's disease (PD). However, none of these studies have employed an objective home-based measure of dyskinesia.

**Objectives:**

To evaluate in advanced PD the relationship between ICB and dyskinesia, objectively measured with a wearable device.

**Methods:**

In this cross-sectional study, ICB and other neuropsychiatric symptoms were assessed by means of structured clinical interview and specific screening instruments. Presence and severity of motor fluctuations and dyskinesia were rated with patient's and clinician's based rating instruments. Motor fluctuations and dyskinesia were also measured at home for 5-days using a validated wearable devise, the Parkinson's KinetiGraph™(PKG).

**Results:**

We included 89 subjects with PD (29 females, 62 ± 7 years, disease duration 10.3 ± 4.5), of whom 36 (40%) had ICB. Patients with and without ICB did not differ by presence and severity of dyskinesia measured by clinical scales and PKG. There was no association between the presence of ICB and dyskinesia in the whole sample.

**Conclusion:**

Our data suggest that ICB and dyskinesia are common but unrelated disorders in advanced PD.

## Highlights

• Patients with and without impulsive compulsive behaviour did not differ for dyskinesia. .
• There was no association between ICB and dyskinesia in the whole sample. .
• ICB and dyskinesia are common but unrelated disorders in PD.

## 1 Introduction

Impulsive compulsive behaviour disorders (ICB) and levodopa induced dyskinesia (LID) are common and disabling complications of Parkinson's disease (PD) which result from the interplay between dopaminergic medications and the ongoing neurodegenerative process [1].

It has been suggested that ICB and LID might have a common pathophysiological basis, given that they share several risk factors and co-occur in some patients [2]. However, experimental and clinical studies to test this hypothesis are limited [3,4]. Here, we aimed to explore the relationship between ICB and LID in a sample of advanced PD patients, in whom we systematically assessed ICB and dyskinesia using reliable instruments: a clinical semi-structured interview with both the patient and carer for the diagnosis of ICB and a wearable device, which provides an ecological objective measure of dyskinesia at home.

## 2 Patients and methods

Consecutive patients with a diagnosis of PD according to MDS Clinical criteria [5] attending the clinic for advanced therapies at St George's University Hospital were recruited. They were referred because they had motor fluctuations and/or dyskinesia impacting on their quality of life. Exclusion criteria were inability to give informed consent and presence of dementia as per clinical assessment according to DSM-V criteria.

We collected the following demographic and clinical characteristics: age, gender, disease duration, information on PD medications, which were then converted in total levodopa equivalent daily dose (LEDD) [6].

Severity of parkinsonism was rated by means of the Unified PD Rating Scale (UPDRS) parts III and IV. The presence of past and active ICB was assessed with a semi-structured interview. The semi-structured interview was conducted by a neurologist and neuropsychologist with the patient and the care-partner. The semi-structured interview was based on DSM-IV-TR criteria for impulse control disorders and explored all ICBs including pathological gambling, compulsive buying, compulsive sexual behaviour, binge eating. We also assessed the presence of punding and related disorders, and compulsive use of dopaminergic medications. The Questionnaire for Impulsive-Compulsive Disorders in Parkinson's Disease–Rating Scale (QUIP-RS) was employed to rate the severity of ICB. Psychiatric symptoms and trait impulsivity were evaluated by means of Hamilton depression rating scale, Hamilton anxiety rating scale, Apathy evaluation scale and Barratt impulsivity scale (BIS-11).

The Unified Dyskinesia Rating Scale (UDRS) part III “Objective Impairment” was used to assess dyskinesia in clinic [7]. Seven anatomical areas are rated for severity of dyskinesia or dystonia with the highest score for the four tasks recorded as the final score for each body region. The score ranges from 0 to 28.

We used a wearable device, the Parkinson's KinetiGraph™ system (PKG® or Personal KinetiGraph™) to objectively evaluate the presence and severity of dyskinesia at home [8]. The Parkinson's KinetiGraph™ system (PKG® or Personal KinetiGraph™ as it is known in the USA) is an accelerometery-based system for automated assessment of dyskinesia and bradykinesia. This system has two algorithms that every 2min, provide a score of the likelihood of movements being either dyskinetic or bradykinetic. The PKG was sent at the patient's house. Patients wore it for 5 consecutive days for 24 h on the wrist of the most affected side. The device was programmed to vibrate and alert the subject when a dose of levodopa was due. The patient confirmed the actual time when each dose was taken by placing their thumb on a sensor zone on the device face. When the recording was completed, data were downloaded and analysed by proprietary algorithms. The PKG logger records accelerometery data in 3 axes continuously over 5 days. The algorithm operates on each 2 min. For this study, we retrieved the median bradykinesia (mBKS) and dyskinesia (mDKS) scores [8] (supplementary material). The median dyskinesia score (mDKS) is the median of the 2 min scores recorded between the periods of 09:00–18:00 for each of the five days. We used the mDKS to classify patients in PD and dyskinesia using the following cut-off: 4.5–7 as mild 7–15 moderate and >15 as severe.

All patients provided written informed consent to participate following the Declaration of Helsinki. The research ethics board approved the study (IRAS number 259146, approved on 12/08/2019 by HRA and Health and Care Research Wales).

### 2.1 Statistical analysis

After checking for normal distribution of the variables, groups comparisons for demographic and clinical data were performed either by t-tests, Mann-Whitney test or Kruskal–Wallis test by ranks as appropriate.

Spearman correlation analysis were employed to evaluate the correlation between severity of ICB (QUIP scores) and dyskinesia (UPDRS-IV dyskinesia sub-score, UDRS, mDKS).

Descriptive statistic is shown as mean and standard deviation (SD), or median and interquartile range (IQR).

SPSS 28 software was used for all statistical analyses. All tests were two-sided with a level of significance set at p < 0.05.

## 3 Results

Out of 145 subjects with PD referred to our centre for advanced therapies, we enrolled 88 subjects (29 females) who consented to participate and wore the PKG for 5 days. They had a mean age of 62.0 ± 7 years and a mean disease duration of 10.3 ± 4.5 years. Thirty-six patients had at least one active ICB (40%). Ten patients had a single ICB, 26 had more than one ICB. There was no significant difference in gender distribution of PD with and without ICB (chi = 0.17, p = 0.6) and of PD with and without dyskinesia (chi = 0.6, p = 0.4). Twenty-six (29%) patients had severe dyskinesia as per mDKS.

**Fig. 1:**
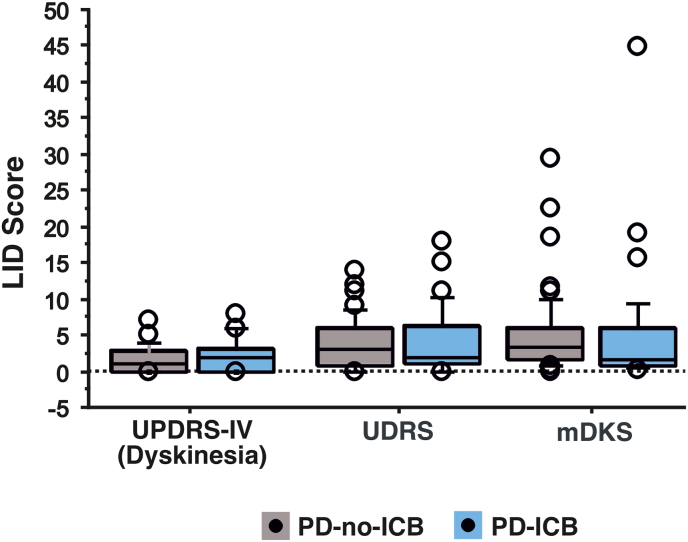
There was no difference between PD with and without impulsive compulsive behaviour (ICB) for presence and severity of dyskinesia measured by UPDRS-IV, Unified Dyskinesia Rating Scale (UDRS) and median dyskinesia score (mDKS), obtained by PKG.

Table 1 and Fig. 1 show the comparisons between PD with active ICB (PD-ICB) and PD without ICB (PD-no-ICB). PD-ICB were more depressed, more anxious and more apathetic than PD-no-ICB. Presence and severity of dyskinesia was similar in the two groups, both when measured with patients' questionnaires (UPDRS-IV total and dyskinesia sub-score) and the clinician's rating scale for dyskinesia (UDRS). PKG analysis confirmed these findings, by showing similar mDKS between PD-ICB and PD-no-ICB. Groups were also comparable for severity of motor symptoms in clinic (UPDRS-III, ON and OFF medications) and daytime bradykinesia at home (mBKS).

**Table 1:** Comparison between PD patients with and without impulsive compulsive disorders.

	PD-no-ICB	PD-ICB	p value
Age (years)	63.3 ± 7.4	59.8 ± 5.7	0.02
Disease duration (years)	10.5 ± 4.9	10 ± 3.8	0.9
Total LEDD	1048.1 ± 421.1	1177.2 ± 333.1	0.09
Dopamine-agonists LEDD	217.2 ± 163.4	174.0 ± 144.3	0.2
Total ICD score	3.1 ± 4.7	15.5 ± 8.2	<0.0001
Total QUIP-RS Score	5.3 ± 7.2	30.9 ± 13.2	<0.0001
BIS-11	44.5 ± 22.6	59.8 ± 21.4	<0.0001
Hamilton Depression Rating Scale	5.6 ± 4.6	10.7 ± 6.4	0.0001
Hamilton Anxiety Rating Scale	6.9 ± 6.7	13.5 ± 7.9	0.0001
Apathy evaluation scale	8.8 ± 5.7	14.6 ± 9.2	0.004
UPDRS III - OFF	38.2 ± 23.2	44.5 ± 19.1	0.2
UPDRS III - ON	17.0 ± 13.3	20.1 ± 12.1	0.2
UPDRS IV	5.6 ± 2.9	7.0 ± 3.8	0.1
UPDRS IV (LID score)	1.5 ± 1.7	2.0 ± 2.3	0.5
UDRS	3.7 ± 3.5	4.0 ± 4.6	0.9
mBKS	25.7 ± 8.3	26.5 ± 7.5	0.4
mDKS	4.6 ± 5.4	4.6 ± 8.3	0.1

Abbreviations: BIS-11: Barratt Impulsivity Scale; ICD: impulse control disorders; ICB: impulsive compulsive behaviour disorders; LEDD: levodopa equivalent daily dose; LID = levodopa induced dyskinesia; mBKS = median bradykinesia score; mDKS = median dyskinesia score; QUIP-RS: Questionnaire for Impulsive-Compulsive Disorders in Parkinson's Disease-Rating Scale; UDRS: Unified Dyskinesia Rating Scale, UPDRS: Unified Parkinson's Disease Rating Scale. Values are means ± Standard deviation.

Severity of ICB by QUIP-ICD and QUIP-RS total score was not correlated to severity of dyskinesia (UPDRS-IV dyskinesia sub-score, UDRS, mDKS) (Supplementary Table 1).There was a significant correlation between mDKS and UDRS (rho = 0.46, p=<0.001) and UPDRS IV dyskinesia sub-scores (rho = 0.26, p = 0.01) (Supplementary Table 1)

## 4 Discussion

We did not find an association between dyskinesia and ICB in people with advanced PD. Moreover, severity of dyskinesia was not related to severity of ICB. These results were strengthened by having objectively measured dyskinesia at home with a wearable device.

Up to 60% of people with PD lack awareness of involuntary movements such as dyskinesia [9]. In general, the severity of LID assessed in clinic might not reflect the magnitude of involuntary movements experienced at home, and to minimize this bias, we used a wearable device that recorded patients’ involuntary movements for five consecutive days. The validity of this measure has been demonstrated in previous studies [8] and it is confirmed in our sample by the significant association between the PKG scores and the clinical assessment by the neurologist (UPDRS-IV and UDRS).

Another strength of our study is that the diagnosis of ICB was based on a semi-structured clinical interview which involved the caregiver and was based on DSM-IV-TR criteria for impulse control disorders. Although the QUIP-RS is a recommended screening instrument for the diagnosis of ICB, the clinical interview remains the best tool to identify these behavioural disturbances whose clinical spectrum is not fully covered by QUIP-RS [10]. Accordingly, self-rating scales such as QUIP-RS may suffer from patients’ lack of awareness or tendency to hide or minimize these problems [10].

The association between ICB and dyskinesia has been investigated only in a few cross-sectional studies, with conflicting results [3,4,11,12]. In the Dominion study, patients with multiple ICBs had more severe dyskinesia (as per UPDRS IV items 32–33 score) compared to those with single ICB [11]. In the ALTHEA study [3], dyskinesia were evaluated using the UDRS. The UDRS includes an historical section and an objective section [7]. PD-ICB had a higher total UDRS score, but this was determined by a significant higher score of the historical section of UDRS (sub-score for off-dystonia) [3]. Indeed, there was no difference when considering the ‘objective’ sub-score of dyskinesia, a result confirmed also by our study that employed the same rating scale.

Our finding of lack of association between LID and ICB is also in keeping with a recent report [12] that found more frequent LID in subjects without ICB. In this study, PD-no-ICB had a significant longer disease duration compared to PD-ICB. This supports the hypothesis that the two phenomena are not pathophysiologically associated, but they might co-occur as they have in common two risk factors: high LEDD and longer disease duration.

Chronic levodopa treatment, largely in a dose-dependent fashion, is the major contributor for the development of LID, together with disease duration [13]. Similar risk factors are associated to ICB, in conjunction with exposure to dopamine agonists in predisposed individuals [1]. A strength of our study is that PD-ICB and PD-no-ICB did not differ by dopamine agonists dose, total LEDD and disease duration. This ensured that the two groups were similar for variables commonly associated to ICB or LID and explains the difference of our results compared to previous research [4].

One of the limitations of our study is the lack of people in the early stages of PD. ICB may occur in up to 20% in subjects with a mean disease duration of 2.6 years) [14] and dyskinesia in 13–40% within the first 2.5–3.5 years [13]. Yet, only a longitudinal study in de novo PD might definitely clarify whether these two phenomena are associated.

We believe that our study captured in an objective ecological manner dyskinesia and ICBs in a cohort of people with advanced PD treated with a high dopaminergic daily dose. Our results suggest that, although the risks of both LID and ICBs are higher in people treated with higher dopaminergic daily dose, the underlying pathophysiological mechanisms mediating the expression of these complications are different. This is also supported by neurophysiological studies showing distinct physio-markers for dyskinesia and ICB, when recording neural activity from the sub-thalamic nucleus and the cortex in PD patients undergoing Deep Brain Stimulation [15].

In conclusion, our data show that levodopa-induced dyskinesia and impulsive compulsive behaviours are common but unrelated disorders in people with advanced PD.

## Ethical compliance statement

Institutional ethics approval was obtained (IRAS number 259146) and approved by the ethical Committee. The study was conducted in accordance with the Declaration of Helsinki. Each participant provided written informed consent before study participation.

We confirm that we have read the Journal's position on issues involved in ethical publication and affirm that this work is consistent with those guidelines.

## Full financial disclosure for the previous 12 months

• Lucia Ricciardi: Research support from UK's Medical Research Council (MRC), Clinical Academic Research Partnerships.
• Francesca Morgante: Research support from NIRH. Speaking honoraria from Abbvie, Medtronic, Boston Scientific, Bial, Merz; Travel grants from the International Parkinson's disease and Movement Disorder Society; Advisory board fees from Merz and Boston Scientific; Consultancies fees from Boston Scientific, Merz and Bial; Research support from NIHR, Innovate UK, Merz and Global Kinetics; Royalties for the book “Disorders of Movement” from Springer; member of the editorial board of Movement Disorders, Movement Disorders Clinical Practice, European Journal of Neurology.
• Mark J Edwards: Honoraria from Merz Pharma and Boeringer Ingleheim. Royalties from the Oxford University Press.
• The other authors do not have any disclosures to declare.

## Authors contribution

Lucia Ricciardi: Conception, Design, Organization, Execution,Writing of the first draft, Review and Critique.

De Angelis A: Execution, Review and Critique.

Chiara Siri: Review and Critique.

Malcom Horne: Design, Review and Critique.

Alison Leake: Execution, Review and Critique.

Dominic Paviour: Execution, Review and Critique.

Pradhan P: Execution, Review and Critique.

Edwards MJ: Review and Critique.

Francesca Morgante: Conception, Design, Organization, Execution, Review and Critique.

## Declaration of competing interest

This study was supported by Global Kinetics.
