# Supplementary material for: Dyskinesia and impulsive compulsive behaviour in Parkinson’s disease are not related: Insights from a study with a wearable sensor
Source: Parkinsonism Relat Disord. 2023 Oct;115:105813. doi: 10.1016/j.parkreldis.2023.105813 (PMC10750257; doi:10.1016/j.parkreldis.2023.105813)
Supplement: Multimedia component 1 [file mmc1.docx]

***Supplementary Methods***

The Parkinson’s KinetiGraph is an accelerometry-based system for automated assessment of dyskinesia and bradykinesia. This system has two algorithms that every two minutes, provide a score of the likelihood of movements being either dyskinetic or bradykinetic (dyskinesia score (DKS) and bradykinesia score (BKS)) (Griffiths et al, 2012). The acceleration recordings are divided into 2-minute epoch and the inputs to the expert system included Mean Spectral Power within bands of acceleration between 0.2 and 4 Hz, peak acceleration and the amount of time within these epochs that there is no movement.

**Median BKS (mBKS):** For each epoch, a bradykinesia score (BKS) was produced (Griffiths et al, 2012). This quantifies low power and acceleration movements that represent bradykinetic movements. The median BKS is the 50th percentile of the BKS for all days that the PKG was worn.

**Median DKS (mDKS)**: For each two-minute epoch, a dyskinesia score (DKS) was produced (Griffiths et al, 2012). This parameter quantifies high acceleration movements that are representative of involuntary movements. The median DKS was the 50th percentile for all days that the PKG was worn. We used the mDKS to classify patients in PD and dyskinesia using the following cut-off: 4.5-7 as mild 7-15 moderate and >15 as severe (Khodakarami et al., 2012. doi: 10.1186/s12984-021-00905-4).

**Supplementary Results**

There was no significant difference in gender distribution in people with and without ICB (chi-square=0.17, p=0.6)

We then looked at differences in number of patients with/without dyskinesia in the 2 groups of PD with ICB and PD without ICB taking into account the age at onset. Indeed, we classified patients as early onset PD (EOPD) if their age at onset was <50 and patients with late onset PD (LOPD) if age at onset was >50. We conducted two within group analyses: one in EOPD and one in LOPD and there was no significant difference in presence/severity of dyskinesia in people with and without ICB both in the group of YOPD (N=30; chi-square=0.05, p=1) and in the group of LOPD (N=52, chi-square=1.8, p=0.2).

*Supplementary Table 1:* Spearman Correlation analysis between severity of ICB and dyskinesia

|  | QUIP-RS Total Score | UPDRS IV dyskinesia sub-score | RDRS | mDKS |
| --- | --- | --- | --- | --- |
| QUIP ICD score | **rho= 0.948, p=<0.001** | rho= -0.02, p=0.8 | rho= -0.1, p=0.3 | rho= -0.19, p=0.07 |
| QUIP-RS Total Score |  | rho= 0.007, p=0.9 | rho= -0.03, p=0.7 | rho= -0.17, p=0.1 |
| UPDRS IV dyskinesia sub-score |  |  | **rho= 0.71, p=<.001** | **rho= 0.26, p=0.01** |
| RDRS |  |  |  | **rho= 0.46, p=<0.001** |

Abbreviations: ICD: impulse control disorders; QUIP-RS: Questionnaire for Impulsive-Compulsive Disorders in Parkinson's Disease-Rating Scale; RDRS: Rush Dyskinesia Rating Scale, UPDRS: Unified Parkinson’s Disease Rating Scale
